# Phlegmonous Abscess

**Published:** 1891-02

**Authors:** Chas. H. Holland

**Affiliations:** Chattanooga, Tenn.


### PHLEGMONOUS ABSCESS.

BY CHAS. H. HOLLAND, M. D., CHATTANOOGA, TENN.

Read at Tri-State Medical Association, Oct. 1890, Chattanooga, Tenn.

On June 4th, I was called to see H. G. J., male, age 25 years, height 6 ft., weight 648 pounds. I saw him early in the morning, and about 11 o'clock p. m. He was suddenly seized with a severe chill while riding on the cars, lasting him for several hours, during which time he was totally unconscious of anything that transpired, and questioning him, I found he was totally ignorant of any illness whatever coming upon him. He complained of severe pains in the head and limbs, more noticeably in the right leg, about half way between the knee and ankle joint. Upon examination of the limb I found considerable odema on the points above mentioned. I noticed a small protuberance, somewhat circumscribed in character, also considerable discoloration of the skin and small lines running in different directions, and of a dark red color. I gave him a hypodermic of morphia and atropia. Ordered him to elevate his limb slightly, and to apply a poultice of flax seed meal and hops to the parts affected, renewing it every three or four hours until I returned. I also left him the following: ℞ Sulph. quinæ, gr. xxx., Acetanelid, gr. xxiv, Sulph. morphia, gr. iv, made into eight capsules and take one every two or three hours as the case required. On the morning of the 5th I found him resting easy, and having slept some during the night. On removing the poultice I found considerable effusion, and at once I determined to make a free incision, and drew about 1-2 gallon of pus from the limb and ordered the treatment to be continued. On the morning of the 6th I found considerable effusion on the inner side of the limb, and about two or three inches from where I first made the incision; I concluded to make another free opening, and drew out about one quart of pus. I ordered him to continue the treatment, and in addition to the former treatment to take 30 gtt. of the tinct. ferri. chloridi. in a wine glass of water three times per day, one hour after each meal. On the morning of the 7th I saw him with my friend, Dr. W. M. H. Bryan. We did not think it necessary to make any change in the treatment. We saw him again on the next evening and found him resting easy. Pulse quiet and temperature normal. By this time the swelling had begun to disappear considerably, and he could begin to move his limb about on the bed, and also work his toes slightly. I never saw him any more until the evening of the 11th, and found him sitting up in bed; the swelling had almost totally disappeared. I ordered him to discontinue all treatment with the exception of the tinct. ferri chloridi., 30 gtt. night and morning in water, instead of three times per day, as before directed. On the morning of the 14th I found him sitting up in a chair and able to walk about with the aid of a crutch; he informed me that he had received a telegram calling him to Cleveland, Ohio, and that he would be compelled to leave on the evening train. He was assisted to the train and left for the above named place. I have not heard from him since. Now, gentlemen, I want you to tell me if my diagnosis and treatment was correct in the case, and explain to me the reason why he never detected this trouble until he was seized with a chill, and tell me under what conditions a man can have pus formed in the above mentioned quantities until so short a time before it has to be evacuated, for he stated that he was well, and did not know anything about the matter whatever until he was seized with the chill.

